# Evaluation of Baloxavir Marboxil and Peramivir for the Treatment of High Pathogenicity Avian Influenza in Chickens

**DOI:** 10.3390/v12121407

**Published:** 2020-12-08

**Authors:** Augustin Twabela, Masatoshi Okamatsu, Keita Matsuno, Norikazu Isoda, Yoshihiro Sakoda

**Affiliations:** 1 Laboratory of Microbiology, Department of Disease Control, Faculty of Veterinary Medicine, Hokkaido University, Sapporo 060-0818, Japan; ttaugushahu@vetmed.hokudai.ac.jp (A.T.); okamatsu@vetmed.hokudai.ac.jp (M.O.); nisoda@vetmed.hokudai.ac.jp (N.I.); 2 Virology Service, Central Veterinary Laboratory of Kinshasa, Ministry of Fisheries and Livestock, Kinshasa I/Gombe 012, Democratic Republic of the Congo; 3 International Collaboration Unit, Research Center for Zoonosis Control, Hokkaido University, Sapporo 011-0020, Japan; matsuk@czc.hokudai.ac.jp; 4 Unit of Risk Analysis and Management, Research Center for Zoonotic Control, Hokkaido University, Sapporo 011-0020, Japan

**Keywords:** baloxavir marboxil, peramivir, treatment, high pathogenicity avian influenza, chicken model

## Abstract

Control measures in the case of high pathogenicity avian influenza (HPAI) outbreaks in poultry include culling, surveillance, and biosecurity; wild birds in captivity may also be culled, although some rare bird species should be rescued for conservation. In this study, two anti-influenza drugs, baloxavir marboxil (BXM) and peramivir (PR), used in humans, were examined in treating HPAI in birds, using chickens as a model. Chickens were infected with H5N6 HPAI virus and were treated immediately or 24 h from challenge with 20 mg/kg BXM or PR twice a day for five days. As per our findings, BXM significantly reduced virus replication in organs and provided full protection to chickens compared with that induced by PR. In the 24-h-delayed treatment, neither drug completely inhibited virus replication nor ensured the survival of infected chickens. A single administration of 2.5 mg/kg of BXM was determined as the minimum dose required to fully protect chickens from HPAI virus; the concentration of baloxavir acid, the active form of BXM, in chicken blood at this dose was sufficient for a 48 h antiviral effect post-administration. Thus, these data can be a starting point for the use of BXM and PR in treating captive wild birds infected with HPAI virus.

## 1. Introduction

High pathogenicity avian influenza (HPAI) is one of the most devastating avian viral diseases. The infection caused by the high pathogenicity avian influenza virus (HPAIV) induces an acute disease with high mortality reaching 100% in chickens [1,2]. Aside from chickens, HPAI also affects other poultry populations such as ducks, turkeys, and quails [3]. Although aquatic wild birds form the natural reservoir of HPAIV [4,5], other wild bird species have been reported to be susceptible to HPAIV infection, which can sometimes become fatal [6,7]. Therefore, it is necessary to not only protect poultry but also wild bird species from HPAIV infection.

HPAI vaccination among chickens and domestic ducks has been implemented as a preventive measure and infection control in some countries [8,9]; however, stamping out the affected population has remained to be the global standard recommendation [10]. Given that the vaccines must be frequently updated due to antigenic changes of viruses occurring in each epidemic [11,12], the vaccine may also lead to the silent spread of HPAIV with asymptomatic infections [13]; vaccination against HPAI is prohibited for use in poultry in many countries [8,14]. Active surveillance of wild birds and the improvement of biosecurity in farms are also performed to monitor and prevent HPAI in addition to stamping out [10]. Wild birds in captivity, such as birds in zoos, may need to be covered by these strategies as well because of their potential susceptibility to HPAIV infection [7].

To protect captive wild birds at risk from HPAIV infection in the zoo or sanctuary, both stamping out and emergency vaccination are conducted [6,15]. However, in the case of valuable captive birds, such as rare species and breeds, culling may not be an option for conservation and economical purposes. Thus, additional approaches are needed in order to treat birds infected with HPAIV using antiviral drugs. Anti-influenza drugs have been intensively developed for human treatment [16,17]. However, only few studies have assessed their effectivity on avian species; for instance, oseltamivir, a pro-drug for neuraminidase inhibition, has been evaluated for its preventative and therapeutic effects against avian influenza virus (AIV) infection in poultry. However, several limitations were noticed in these studies, such as a lack of clinical signs in chickens when challenged with a low pathogenic avian influenza virus [18]; presence of virus growth and embryo mortality but not clinical signs as can it be observed in adult birds, when eggs were used for HPAIV infection [19,20]; and partial protection of chickens infected with HPAIV [21]. Furthermore, the metabolism of oseltamivir in bird species was not evaluated for the production of an active drug form for influenza treatment as seen in humans or mice.

In this study, two anti-influenza drugs used in humans were evaluated for the potential treatment of HPAI in rare birds. This included baloxavir marboxil (BXM), a novel cap-dependent endonuclease inhibitor that acts as a pro-drug, and peramivir (PR), a neuraminidase inhibitor used as a control because its active form has a direct anti-influenza effect for the treatment of AIV infection. The antiviral efficacy of these drugs was evaluated using the chicken model, which demonstrated severe HPAI infection, and the metabolism of BXM in both chickens and Pekin ducks was assessed to determine the effective dosage that can be applied for other bird species.

## 2. Materials and Methods

### 2.1. Antiviral Drugs

A 20 mg tablet of BXM, was used for oral administration; BXM is metabolized in the liver to baloxavir acid (BXA), which is the active form of BXM. 10 mg/mL solution of PR, was administered via intramuscular injection. Both drugs were purchased from Shionogi & Co., Ltd., Osaka, Japan. For drug administration to birds, a tablet of BXM was crushed into powder and was further dissolved in 2 mL of sterile phosphate-buffered saline (PBS); then the volume was calculated accordingly (e.g., 1 mL for 10 mg, 100 µL for 1 mg, 50 µL for 0.5 mg and so on). For PR, 1 mL containing 10 mg was administered.

### 2.2. Virus

HPAIV A/black swan/Akita/1/2016 (H5N6) (A/BS/Akita/1/16) viral strain, isolated from a black swan in a zoo in Japan [22], was used as a challenge strain in chickens. The virus was propagated in 10-day-old embryonated chicken eggs, and the infectious allantoic fluid was used for infection. The inoculum contained 10^5.3^ egg infectious doses (EID_50_), which is equivalent to 10 chicken lethal doses per 100 µL (10 CLD_50_/100 µL).

### 2.3. Animal Experiments

For all challenge experiments, 6-week-old chickens (*Gallus gallus domesticus*, Julia strain; 0.5 kg body weight, obtained from Hokkai Starchick, Hokkaido, Japan) were used in this study. The chickens were housed in conventional conditions without vaccination and were tested as free of AIV antibodies by hemagglutination inhibition (HI) test [23] against the challenge A/BS/Akita/1/16 virus. Three experiments were carried out to mimic the different field conditions of infection during an HPAI outbreak and subsequent treatment.

#### 2.3.1. Experiment 1. Simultaneous Treatment with BXM or PR in the Chicken Model

Chickens were divided into three groups of eight birds each (BXM, PR, and no treatment as control). The chickens were infected intranasally with 100 µL of the lethal A/BS/Akita/1/16 virus. A dose of 20 mg/kg was immediately administered orally for BXM and intramuscularly for PR. The treatment was repeated every 12 h for 5 days. The 20 mg/kg dose was determined based on the dose of 1 mg/kg used in humans for clinical treatment [24]; however, because of the high dose (10 CLD_50_) of the challenge virus, the doses were adjusted accordingly.

#### 2.3.2. Experiment 2. Delayed Treatment with BXM or PR in the Chicken Model

Chickens were divided the same as in Experiment 1. However, the treatment for all chickens started 24 h after the infection when the first clinical signs such as depression and lack of appetite appeared. Drugs were then administered twice a day for 5 days.

#### 2.3.3. Experiment 3. Assessment of the Minimum Dose of BXM in the Chicken Model

A range of five doses (0.1, 0.5, 2.5, 12.5 and 62.5 mg/kg) was prepared to determine the minimum dose of BXM at a single administration; this because the dose of 20 mg/kg used in Experiments 1 and 2 was higher compared with that used for human and mouse administration. Therefore, chickens were divided into six groups (eight birds each) depending on the dosage, with one group serving as the control; four chickens were assigned for organ sampling at 3 days post-infection (dpi), and the remaining chickens were assigned for clinical observation for 14 days. Chickens were infected with 100 µL of the challenge virus, and a single dose of BXM was orally administered immediately after the infection. In all three experiments, organ samples including the trachea, lung, kidney, colon, and brain were collected at 3 dpi from four out of eight chickens in each group for virus recovery. The remaining chickens were assigned for 14 days of clinical observation.

In addition to sample collection from organs at 3 dpi, swabs were collected from the surviving chickens at 0, 3, 5, 7, and 14 dpi in the clinical observation groups. Briefly, tracheal and cloacal swabs were collected and put separately in 2 mL of viral transport medium (VTM) containing minimum essential medium (MEM: Nissui Pharmaceutical, Tokyo, Japan), 10,000 U/mL penicillin G (Meiji Seika Pharma, Tokyo, Japan), 10 mg/mL streptomycin (Meiji Seika Pharma, Tokyo, Japan), 0.3 mg/mL gentamicin (TAKATA Pharmaceutical Co., Ltd. Saitama, Japan), 250 U/mL nystatin (Sigma-Aldrich, St Louis, MO, U.S.A.), and 0.5% BSA fraction V (Roche, Basel, Switzerland). After vortexing and centrifugation, the supernatant was kept in aliquots at −80 °C until use. At 14 dpi before euthanasia, blood was collected from the surviving chickens for antibody response assessment in serum against the challenge virus by HI test [23].

#### 2.3.4. Assessment of BXA and PR Kinetics in Chickens and Ducks

To assess the metabolism of BXM and the kinetics of BXA and PR for the determination of the appropriated dosage in bird species, three chickens were assigned for each drug, and a single dose of 20 mg/kg was administered orally and intramuscularly for BXM and PR, respectively. For BXM, the blood was collected at 0, 8, 24, and 48 h post-administration (hpa), whereas for PR it was collected at 0, 0.5, 2, and 8 hpa. The duration of blood sampling was different for BXM and PR treatment given the long plasma half-life previously reported for BXM in humans [25] and the shorter plasma half-life reported for PR in mice [26] and humans [27].

To assess the BXM metabolism and BXA blood concentration in different bird species, Pekin ducks were used in the study as well. Three 6-week-old chickens and three 4-week-old Pekin ducks (*Anas platyrhynchos domesticus*, Cherry Valley; obtained from Takikawa Shinseien, Hokkaido, Japan) received a single dose of 2.5 mg/kg of BXM; blood samples were then collected at 0, 8, 24, 48, 72, 96, and 120 hpa.

Blood was collected in heparinized tubes, and plasma was obtained after centrifugation at 3000 rpm for 5 min. For the BXA stability, 3 µM of dichlorovinyl dimethyl phosphate (Wako Chemicals, Tokyo, Japan) was added to the plasma before storage at −80 °C for further analysis. The BXA and PR plasma concentration was determined using a validated liquid chromatography–tandem mass spectrometry method at Sumika Chemical Analysis Service, Ltd., (Osaka, Japan) as previously described [28]. The drug plasma concentration was calculated as a mean standard deviation (SD) of three individual values plotted in the graph.

#### 2.3.5. Virus Recovery in Organs and Shedding in Swabs of Chickens

To evaluate the virus replication in infected chickens after the treatment, organs collected at 3 dpi in all three challenge experiments were homogenized using a Multi-Beads Shocker (Yasui Kikai, Osaka, Japan) to make a 10% (*w*/*v*) of suspension in VTM. Viral infectivity was assessed based on the cytopathogenic effect (CPE) observed in the monolayer of Madin–Darby canine kidney (MDCK) cells maintained in MEM supplemented with 10% nonimmobilized fetal calf serum (FCS; SAFC Biosciences, Lenexa, KS, USA), 0.3 mg/mL L-glutamine (Wako Chemicals, Tokyo, Japan), 100 U/mL penicillin G, 0.1 mg/mL streptomycin, and 8 µg/mL gentamicin; cells were then incubated at 37 °C in 5% CO_2_. Briefly, tenfold dilutions of the organ supernatant with VTM were performed in an FCS-free MEM; each dilution was inoculated in 4 wells and incubated for 1 h at 37 °C. Cells were later washed twice with PBS, and 100 µL of FCS-free MEM was added and incubated for 72 h at 37 °C in 5% CO_2_. The 50% tissue culture infectious dose (TCID_50_) was calculated according to Reed and Muench [29], which was set as the viral titer of the sample.

To measure the virus shedding of chickens after infection and treatment with BXM, tenfold dilutions of swab suspension in VTM were each inoculated into four 10-day-old chicken embryonated eggs obtained from a conventional flock tested free of AIV. After a 48-h incubation at 35 °C, the hemagglutination test using 1% chicken red blood cells [23] was performed to assess the infectivity titer calculated as EID_50_ [29].

### 2.4. Statistical Analysis

To compare the virus replication between the treatment groups, one-way analysis of variance with Tukey test was performed using Statistical Package for the Social Sciences (SPSS) version 20.0 (IBM Corp, Armonk, NY, USA). The difference was considered significant at a *p*-value < 0.05.

### 2.5. Ethical Statement

All animal experiments were performed in the animal biosafety level 3 facility at the Faculty of Veterinary Medicine of Hokkaido University, Sapporo, Japan, with the approval number 18-0090 obtained on 3 March 2019, under the guidelines of the Institutional Animal and Committee of Hokkaido University, certified by the Association for Assessment and Accreditation of Laboratory Animal Care International (AAALAC International) since 2007.

## 3. Results

### 3.1. Effects of Multiple Doses of BXM or PR in the Simultaneous Treatment of HPAIV Infection

**Figure 1:**
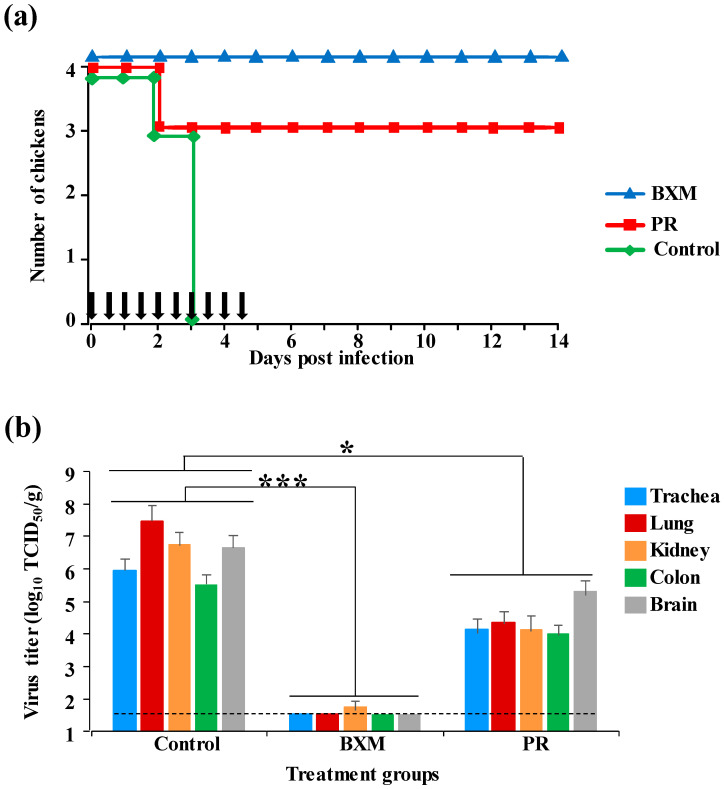
Effects of the simultaneous treatment using multiple doses of baloxavir marboxil (BXM) or peramivir (PR) in chickens infected with a high pathogenicity avian influenza virus (HPAIV), A/BS/Akita/1/16. Eight chickens per group were infected with 10 CLD_50_/100 µL (10^5.3^ EID_50_) of the HPAIV and treated immediately with 20 mg/kg of either BXM or PR; the untreated group was assigned as the control. (**a**) Survival of half the infected birds was followed until 14 days post-infection (dpi); vertical black arrows indicate the treatment schedule. (**b**) Virus recovery from organs collected at 3 dpi from four chickens in the control and each treatment group was assessed in monolayer Madin–Darby canine kidney (MDCK) cells. The viral titer calculated as the average (*n* = 4) of each organ in the treatment group was compared to that of the control group. The black dotted line indicates the detection limit (10^1.5^ TCID_50_/g), whereas the bar indicates the standard deviation. (*, *p* < 0.05; ***, *p* < 0.001).

To evaluate the protective effect of BXM or PR in chickens following HPAIV infection, bird survival was monitored, and viral replication in the organs was measured. No deaths were recorded in BXM; one chicken died at 2 dpi in PR group with treatment at 20 mg/kg, whereas all chickens died by 3 dpi in the control group (Figure 1a). Viral replication in the organs of the BXM-administrated chickens has been noticed to decrease compared with that in the control group (*p* < 0.001); however, in PR group, viral replication was noticeably higher even though the titers did not reach those observed in the control group (Figure 1b). As per our findings, BXM treatment was able to suppress viral replication, enabling the chickens to survive infection, whereas only partially protective efficacy was observed with PR treatment because of insufficient inhibition of viral replication.

### 3.2. Effects of Multiple Doses of BXM or PR in the Delayed Treatment of HPAIV Infection

**Figure 2:**
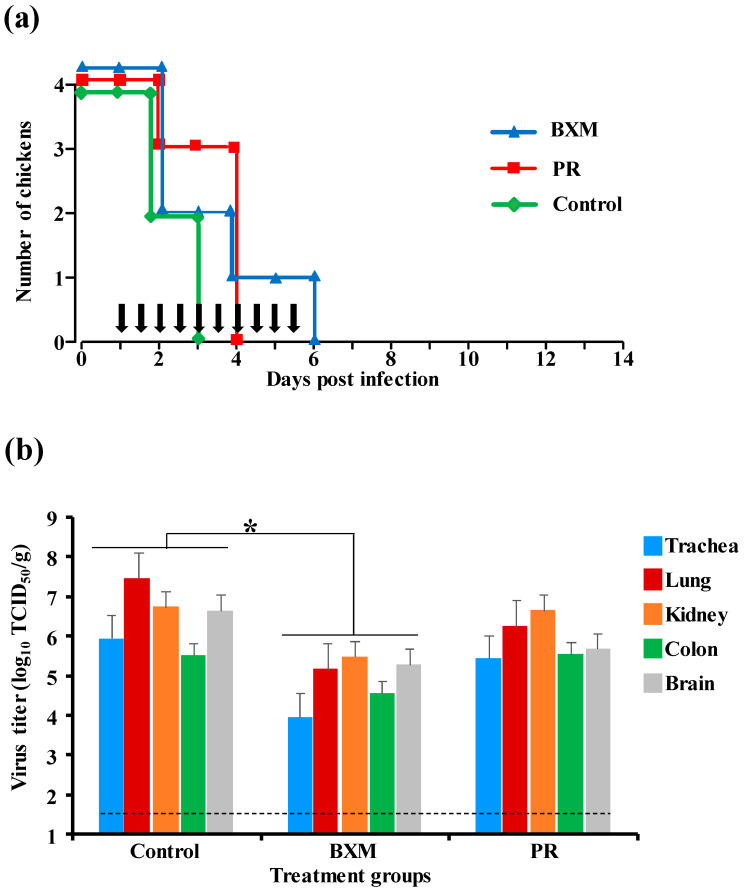
Effects of delayed treatment with multiple-dose administration of BXM or PR in chickens infected with HPAIV, A/BS/Akita/1/16. Eight chickens per group were infected with 10 CLD_50_/100 µL (10^5.3^ EID_50_) of the HPAIV and treated with 20 mg/kg of either BXM or PR for 24 h to 5 days post-infection; the untreated group was kept as the control. (**a**) Survival and clinical signs of half the infected birds was followed until 14 dpi; vertical black arrows indicate the treatment schedule. (**b**) Virus recovery from organs collected at 3 dpi from four chickens of each group was assessed in monolayer MDCK cells. The viral titer calculated as the average (*n* = 4) of each organ in the treatment group was compared to that of the control group; bar indicates the standard deviation. (*, *p* < 0.05).

The simultaneous treatment of chickens with either BXM or PR demonstrated the protective effects of both two drugs; a further experiment was carried out to assess the protective potential of both of these drugs starting at the notification of clinical signs at 24 h post-infection, which mimics the field situation of an HPAI outbreak. All the chickens in the three groups (BXM, PR, and control) succumbed to HPAI infection. However, in the BXM group, one chicken survived up to 6 dpi, whereas in the PR group, three chickens survived until 4 dpi, compared with those in the control group, which all had died by 3 dpi (Figure 2a). Virus replication in chicken organs at 3 dpi was significantly lower in the BXM-treated group than that in the control group (*p* < 0.05), although there was no significant difference between the PR and control group (Figure 2b). Despite the mortality observed in the three groups, BXM treatment had prolonged the survival of one chicken and suppressed viral replication to a greater extent than that compared to using PR.

### 3.3. Protective Effect of a Single Dose of BXM in the Simultaneous Treatment of HPAI in the Chicken Model

**Figure 3:**
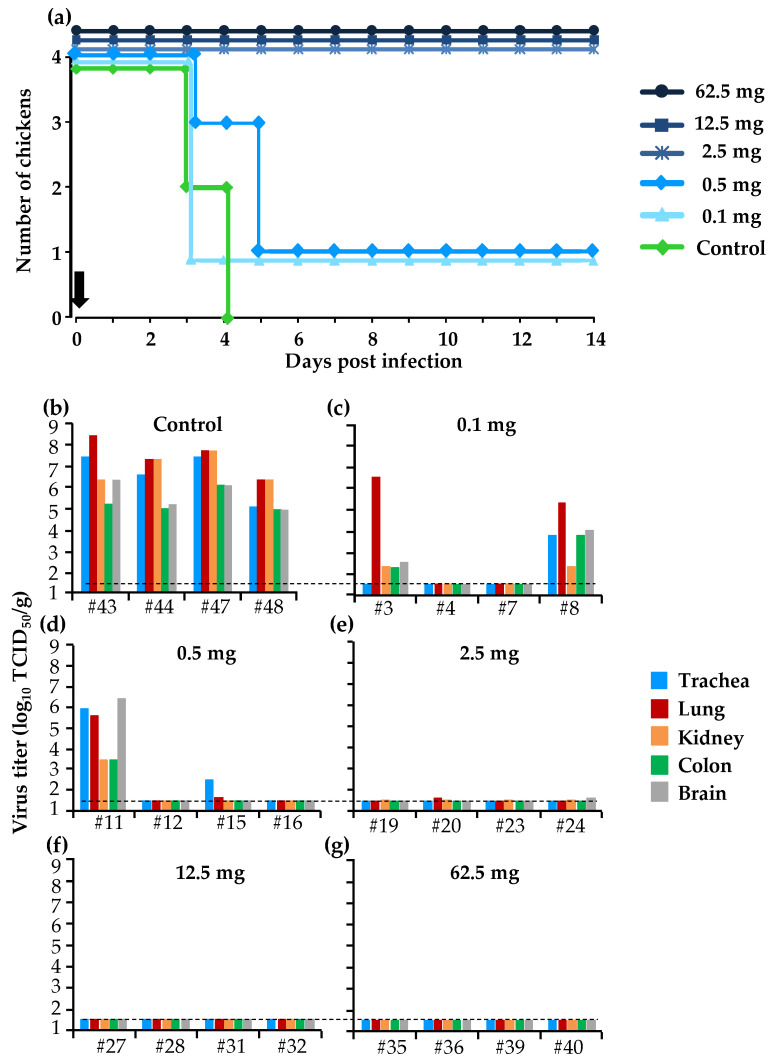
Effects of the simultaneous treatment with a single dose of BXM in chickens. Eight chickens per treatment group were infected with 10 CLD_50_/100 µL (5.3 log_10_ EID_50_) of the HPAIV, A/BS/Akita/1/16, and they were immediately treated with different doses of BXM or untreated to serve as the control. (**a**) Survival of half the birds in each group was monitored until 14 dpi; the vertical black arrow indicates the treatment schedule. (**b**–**g**) Viral recovery from organs collected at 3 dpi from four chickens was assessed in MDCK cells; the black dotted line indicates the detection limit (1.5 log_10_ TCID_50_/g).

Multiple administrations of BXM (20 mg/kg) has provided complete protection in chickens against the lethal infection with HPAIV, whereas the same dose of PR has only partially achieved slight inhibition of virus growth (Figure 1a,b). Therefore, we sought to determine the minimum dose that could protect chickens from the lethal infection. When chickens were infected with 10 CLD_50_/100 µL of A/BS/Akita/1/16 followed immediately by a single administration of different doses of BXM (0.1, 0.5, 2.5, 12.5, and 62.5 mg/kg), all the chickens in the groups administered with 2.5, 12.5, and 62.5 mg/kg survived for 14 days. However, the dosage of 0.1 and 0.5 mg/kg BXM only protected one of the four treated chickens (Figure 3a). Viral replication in most of the chicken organs was completely inhibited in chickens treated with 2.5, 12.5, and 62.5 mg/kg BXM (Figure 3e–g), although low virus titer was detected in the lung and brain of two chickens treated with 2.5 mg/kg BXM (Figure 3e). In the 0.1 and 0.5 mg/kg BXM-treated groups, the viral replication was completely inhibited in the organs of two of the four chickens (Figure 3c,d) compared with those in the control group where virus replication was relatively high (Figure 3b).

### 3.4. Kinetics of BXA and PR in Chickens and Ducks

**Figure 4:**
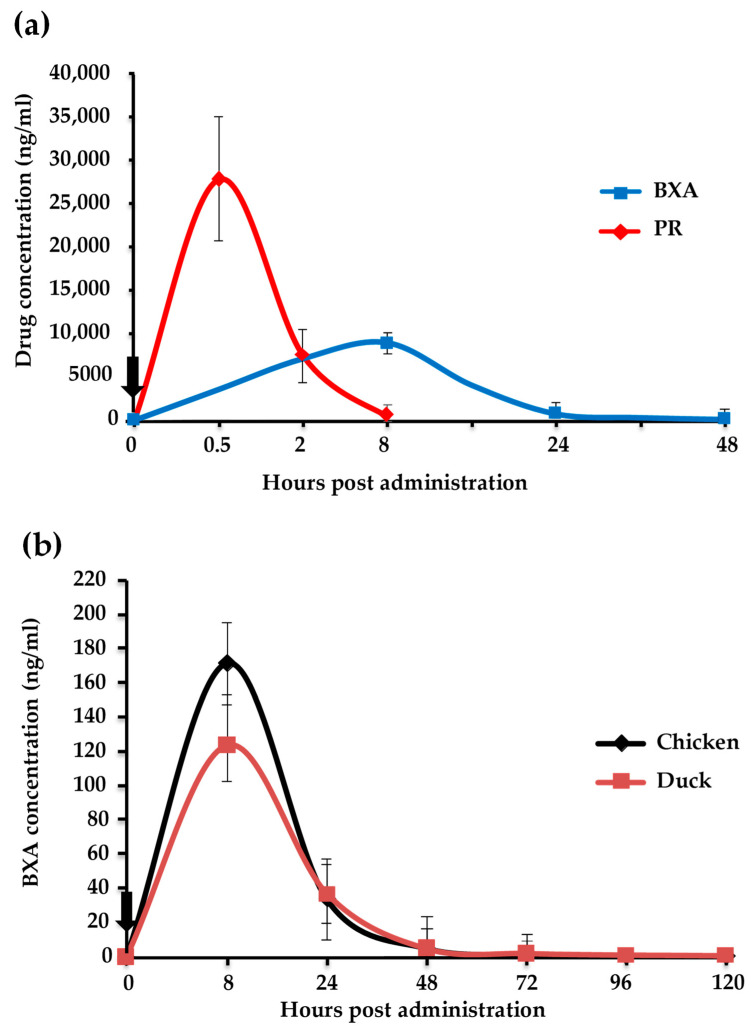
Drug concentration in the blood of chickens and ducks. (**a**) Three chickens per group received 20 mg/kg of BXM or PR. The blood was collected at 0, 8, 24, and 48 h post-administration (hpa) for BXM and at 0, 0.5, 2, and 8 hpa for PR. (**b**) BXA plasma concentration in chickens and ducks administered with 2.5 mg/kg of BXM. Blood was collected at 0, 8, 24, 48, 72, 96, and 120 hpa. The curve shows the mean plasma concentration of BXA and PR at different time points. The bar indicates the standard deviation (*n* = 3). The black vertical arrow indicates the drug administration schedule.

BXM and PR are drugs developed for the treatment of influenza in humans, and there is no available data concerning their dosage and kinetics in avian species. Given that the dosage of 20 mg/kg used in Experiments 1 and 2 was higher compared with that used clinically in humans [30,31], the concentration of the two drugs in blood was measured to adjust for the appropriate dosage for birds. The mean plasma concentration for 20 mg/kg of BXA was 8808.9, 715.3, and 80.4 ng/mL at 8, 24, and 48 hpa, respectively. For PR, the mean plasma concentration was 27,809.7, 7422.9, and 390.8 ng/mL at 0.5, 2, and 8 hpa, respectively (Figure 4a). This relatively high concentration was a consequence of the higher dose of drugs administered to chickens compared with their body weight (20 mg/kg).

After adjusting the BXM dosage to 2.5 mg/kg in chickens, the kinetics of BXA was assessed in chickens and ducks to compare the BXM metabolism in different bird species. In chickens, the maximum plasma concentration (C_max_) was 171.3 ng/mL at 8 hpa, which decreased gradually to 0.14 ng/mL at 120 hpa. In ducks, the C_max_ was 122.8 ng/mL at 8 hpa, which decreased to 0.4 ng/mL at 120 hpa (Figure 4b). No significant difference was observed in the plasma concentration between the two species, indicating that the metabolism of BXM is similar between chickens and ducks.

### 3.5. Virus Shedding and Antibody Response in Chickens

To determine how long the surviving chickens could shed virus following infection and single treatment with different doses of BXM, swabs were collected at 0, 3, 5, 7, and 14 dpi, and these were inoculated in eggs. No virus was recovered from swabs of surviving chickens treated with 0.1, 0.5, 2.5, 12.5, and 62.5 mg/kg. However, high virus titers ranging from 2.7 to 4.2 log_10_ EID_50_/mL were recovered from the swabs collected on the three chickens that had died by 5 dpi in the 0.1 and 0.5 mg/kg treatment groups (Table 1). Furthermore, to ascertain whether the surviving chickens were infected, the antibody response against the challenge virus was examined. The HI titers for all surviving chickens ranged from 32 to 64 HI (Table 1). These results indicate that although infection was established in chickens by inoculation with HPAI virus, viral excretion and clinical signs were predominantly suppressed by BXM activity at a dose of 2.5 mg/kg or higher. Together, chicken survival and virus recovery can indicate that the protective effects of BXM were dose-dependent, and the minimum dose that conferred full protection to chickens from a lethal dose challenge of HPAIV infection is 2.5 mg/kg.

## 4. Discussion

HPAI remains to be a major concern for poultry and wild bird populations. Currently, there is no approved drug for the treatment of HPAIV infection in birds although other preventive measures can be applied, mostly for the poultry populations. To protect captive wild birds from HPAIV infection, studies have been carried out to establish its treatment and prevention using different models. Lee et al. [18], Kaleta et al. [19], and Meijer et al. [21] have evaluated the use of oseltamivir, although several limitations were noticed to apply it efficiently for AI treatment. Other studies have also evaluated the use of poultry vaccines for the prevention of HPAI caused by H5 and H7 virus strains in zoo birds [15,21,32]. However, an effective immune response was not induced in all of the vaccinated birds, which only implies that a proportion of birds remain at risk for HPAIV infection. Additionally, vaccines against AI are usually subtype- or strain-specific, whereas the AI outbreak is not predictable for such a vaccine specificity; in the case of emergency vaccination for a specific AI strain, the birds will still be exposed to infection during the seroconversion period. Moreover, in some countries, vaccine use for AI is prohibited for poultry and in wild bird populations. In the present study, chickens were used, a susceptible avian species to AIV, as an animal model to test for HPAIV infection, which has been known for its high virus replication in the organs and high mortality, mimicking a natural HPAI outbreak in the field. Our model demonstrates that both BXM and PR were efficacious for the protection of chickens in simultaneous treatment, although in the group administered with PR, one out of four chickens died, and virus replication was not completely inhibited. Taken together, these results provide an additional approach to overcome the limitations of vaccination and other traditional measures used to protect captive wild birds in the case of an HPAI outbreak.

Initially, a dose of 20 mg/kg of BXM or PR was administered to chickens. In the simultaneous treatment, the survival of birds and virus titer in organs differed between the BXM and PR groups, with clear beneficial effects by BXM (Figure 1a,b). Moreover, with delayed treatment, even though a net difference was not observed in both survival and the virus recovery, one chicken in the BXM group survived up to 6 dpi and the virus titer was lower compared with those in the control group (Figure 2a). These results indicate that BXM was more effective compared to PR; therefore, BXM was used for further experiments. The dosage of 20 mg/kg to chickens was higher compared to that used in humans [24] and mice; this dose was then adjusted to 2.5 mg/kg, which was determined to be the minimum dose that protected 100% of infected chickens with complete inhibition of virus replication in the organs as well as virus shedding, although the virus was recovered at low titer in organs from two chickens in this treatment group (Figure 3e). This remnant viral population could be due to the slower elimination of the virus from these chickens given that no virus was recovered in the swabs collected at 3 to 14 dpi from four chickens in the same group (Table 1). In summary, although dosage guidelines were provided in this study, it may not be possible to determine the appropriate dosage for each bird species without examining the concentration of BXA in blood. Nevertheless, administering 62.5 mg/kg to chickens, which is approximately 60-fold higher than that used in humans and mice, did not exhibit any side effects; this finding suggests that for treatment purposes, the proposed dosage of 2.5 mg/kg or more can be administered to other bird species in the case of HPAIV infection.

No virus was recovered with swabs collected from chickens treated with 2.5 mg/kg of BXM, although the antibody response revealed that these chickens were infected (Table 1). On the other hand, BXA plasma concentration of BXA for the 2.5 mg/kg dose at 48 h was 4.48 and 4.28 ng/mL in chickens and Pekin ducks, respectively, which is similar to the 6.92 and 6.85 ng/mL plasma concentrations reported to be effective for inhibition of virus replication in humans [25] and mice [33], respectively. This suggests that a single dose of 2.5 mg/kg can prevent virus replication and shedding from infected birds. However, HAIV is reported to persist up to 15 days in the environment after an outbreak, with the possibility of infecting other healthy birds in the flock [34,35,36]. Therefore, it would be necessary to administer four consecutive doses of 2.5 mg/kg at 48 h intervals to an entire flock to maintain the BXA blood concentration above 4.28 ng/mL, which was assumed to have the antiviral effect in chickens in this study; this would ensure full protection for all birds.

In our study, we used an avian origin HPAIV for the challenge experiment and the efficacy of BXM was demonstrated. We cannot minimize the possibility of the circulation of other avian influenza strains potentially resistant to BXM due to some mutations in PA protein as reported previously [31,37].

## 5. Conclusions

In this study, BXM and PR were demonstrated to be effective in treating HPAI virus infection in the simultaneous treatment. However, for the delayed treatment reflecting a real-life field situation, the antiviral efficacy was limited, indicating that early administration would be essential in the case of an HPAI outbreak. The BXM treatment could be the first choice for protecting valuable wild birds from culling or death during an HPAI outbreak, whereas PR as a second choice might be used even though the antiviral effects were limited in this study. Using the chicken and duck models, the administration of 2.5 mg/kg of BXM induced a sufficient plasma concentration of BXA to protect birds from HPAI infection for 48 h. Further studies may be essential to optimize the dosage of BXM for treating HPAI in the wild bird species by evaluating the metabolism of the BXM for a variety of species, despite the similarities observed in chickens and Pekin ducks in our experiments.

## Figures and Tables

**Table 1:** Virus recovery in tracheal and cloacal swabs and antibody response in the serum of the chickens treated with different doses of BXM at single administration.

Treatment (mg/kg)	Chicken ID	Virus Recovery from Swabs on Days Post-Challenge (log_10_ EID_50_/mL) ^a^					HI Titer in Serum ^b^	
		0	3	5	7	14	0	14
		T/C	T/C	T/C	T/C	T/C		
	# 41	–/–	5.2/4.8	†	†	†	<2	†
Control	# 42	–/–	4.9/4.0	†	†	†	<2	†
	# 45	–/–	5.5/4.3	†	†	†	<2	†
	# 46	–/–	5.3/4.5	†	†	†	<2	†
0.1	# 1	–/–	4.2/3.5	†	†	†	<2	†
	# 2	–/–	–/–	–/–	–/–	–/–	<2	64
	# 5	–/–	3.8/2.7	†	†	†	<2	†
	# 6	–/–	4.2/3.5	†	†	†	<2	†
0.5	# 9	–/–	3.4/2.7	†	†	†	<2	†
	# 10	–/–	3.5/3.5	3.7/3.2	†	†	<2	†
	# 13	–/–	3.2/3.0	2.6/3.0	†	†	<2	†
	# 14	–/–	–/–	–/–	–/–	–/–	<2	64
2.5	# 17	–/–	–/–	–/–	–/–	–/–	<2	64
	# 18	–/–	–/–	–/–	–/–	–/–	<2	64
	# 21	–/–	–/–	–/–	–/–	–/–	<2	64
	# 22	–/–	–/–	–/–	–/–	–/–	<2	64
12.5	# 25	–/–	–/–	–/–	–/–	–/–	<2	32
	# 26	–/–	–/–	–/–	–/–	–/–	<2	64
	# 29	–/–	–/–	–/–	–/–	–/–	<2	64
	# 30	–/–	–/–	–/–	–/–	–/–	<2	64
62.5	# 33	–/–	–/–	–/–	–/–	–/–	<2	64
	# 34	–/–	–/–	–/–	–/–	–/–	<2	64
	# 37	–/–	–/–	–/–	–/–	–/–	<2	64
	# 38	–/–	–/–	–/–	–/–	–/–	<2	64

^a^ The virus titer in tracheal and cloacal swab; (#) indicates the identification for each chicken; (–/–) indicates under detection limit (0.5 log_10_ EID_50_/mL) for both T and C swab supernatant.; ^b^ The antibody titer in the serum before and after infection.; (†) indicates that chicken died during the observation period; (T) and (C) indicate tracheal and cloacal swabs, respectively.

## References

[1] Capua I., Alexander D.J. (2004). Avian influenza: Recent developments. Avian Pathol..

[2] de Wit E., Kawaoka Y., de Jong M.D., Fouchier R.A.M. (2008). Pathogenicity of highly pathogenic avian influenza virus in mammals. Vaccine.

[3] Alexander D.J., Parsons G., Manvell R.J. (1986). Experimental Assessment Of The Pathogenicity Of Eight Avian Influenza A Viruses Of H5 Subtype For Chickens, Turkeys, Ducks And Quail. Avian Pathol..

[4] Webster R.G., Bean W.J., Gorman O.T., Chambers T.M., Kawaoka Y. (1992). Evolution and ecology of influenza A viruses. Microbiol. Rev..

[5] Ottis K., Bachmann P.A. (1983). Isolation and characterization of ortho- and paramyxoviruses from feral birds in Europe. Zent. Vet. B.

[6] Oh S., Martelli P., Hock O.S., Luz S., Furley C., Chiek E.J., Wee L.C., Keun N.M. (2005). Field study on the use of inactivated H5N2 vaccine in avian species. Vet. Rec..

[7] Krone O., Globig A., Ulrich R., Harder T., Schinköthe J., Herrmann C., Gerst S., Conraths F.J., Beer M. (2018). White-Tailed Sea Eagle (*Haliaeetus albicilla*) Die-Off Due to Infection with Highly Pathogenic Avian Influenza Virus, Subtype H5N8, in Germany. Viruses.

[8] Peyre M., Fusheng G., Desvaux S., Roger F. (2009). Avian influenza vaccines: A practical review in relation to their application in the field with a focus on the Asian experience. Epidemiol. Infect..

[9] Swayne D.E. (2012). Impact of Vaccines and Vaccination on Global Control of Avian Influenza. Avian Dis..

[10] Swayne D.E., Spackman E. (2013). Current status and future needs in diagnostics and vaccines for high pathogenicity avian influenza. Dev. Biol..

[11] Ohkawara A., Okamatsu M., Ozawa M., Chu D.-H., Nguyen L.T., Hiono T., Matsuno K., Kida H., Sakoda Y. (2017). Antigenic diversity of H5 highly pathogenic avian influenza viruses of clade 2.3.4.4 isolated in Asia. Microbiol. Immunol..

[12] WHO (2017). Antigenic and Genetic Characteristics of Zoonotic Influenza Viruses and Development of Candidate Vaccine Viruses for Pandemic Preparedness.

[13] Gulbudak H., Martcheva M. (2014). A Structured Avian Influenza Model with Imperfect Vaccination and Vaccine-Induced Asymptomatic Infection. B Math. Biol..

[14] Kakogawa M.O.M., Kirisawa R., Asakawa M. (2019). Countermeasures for avian influenza outbreaks among captive avian collections at zoological gardens and aquariums in Japan. Microbiol. Exp..

[15] Philippa J.D.W., Munster V.J., van Bolhuis H., Bestebroer T.M., Schaftenaar W., Beyer W.E.P., Fouchier R.A.M., Kuiken T., Osterhaus A.D.M.E. (2005). Highly pathogenic avian influenza (H7N7): Vaccination of zoo birds and transmission to non-poultry species. Vaccine.

[16] Shie J.-J., Fang J.-M. (2019). Development of effective anti-influenza drugs: Congeners and conjugates—A review. J. Biomed. Sci..

[17] Principi N., Camilloni B., Alunno A., Polinori I., Argentiero A., Esposito S. (2019). Drugs for Influenza Treatment: Is There Significant News?. Front. Med. (Lausanne).

[18] Lee D.-H., Lee Y.-N., Park J.-K., Yuk S.-S., Lee J.-W., Kim J.-I., Han J.S., Lee J.-B., Park S.-Y., Choi I.-S. (2011). Antiviral Efficacy of Oseltamivir Against Avian Influenza Virus in Avian Species. Avian Dis..

[19] Kaleta E.F., Blanco Peña K.M., Yilmaz A., Redmann T., Hofheinz S. (2007). Avian influenza A viruses in birds of the order Psittaciformes: Reports on virus isolations, transmission experiments and vaccinations and initial studies on innocuity and efficacy of oseltamivir in ovo. Dtsch. Tierarztl. Wochenschr..

[20] Tare D.S., Kode S.S., Hurt A.C., Pawar S.D. (2019). Assessing the susceptibility of highly pathogenic avian influenza H5N1 viruses to oseltamivir using embryonated chicken eggs. Indian J. Med. Res..

[21] Meijer A., van der Goot J.A., Koch G., van Boven M., Kimman T.G. (2004). Oseltamivir reduces transmission, morbidity, and mortality of highly pathogenic avian influenza in chickens. Int. Congr. Ser..

[22] Okamatsu M., Ozawa M., Soda K., Takakuwa H., Haga A., Hiono T., Matsuu A., Uchida Y., Iwata R., Matsuno K. (2017). Characterization of highly pathogenic avian influenza virus A(H5N6), Japan, November 2016. Emerg. Infect. Dis..

[23] Afonso C.L., Miller P.J., Grund C., Koch G., Peeters B., Selleck P.W., Srinivas G.B. (2012). The Manual of Diagnosis Tests and Vaccines for Terrestrial Animals.

[24] Hayden F.G., Sugaya N., Hirotsu N., Lee N., de Jong M.D., Hurt A.C., Ishida T., Sekino H., Yamada K., Portsmouth S. (2018). Baloxavir Marboxil for Uncomplicated Influenza in Adults and Adolescents. N. Engl. J. Med..

[25] Koshimichi H., Ishibashi T., Kawaguchi N., Sato C., Kawasaki A., Wajima T. (2018). Safety, Tolerability, and Pharmacokinetics of the Novel Anti-influenza Agent Baloxavir Marboxil in Healthy Adults: Phase I Study Findings. Clin. Drug Investig..

[26] Yun N.E., Linde N.S., Zacks M.A., Barr I.G., Hurt A.C., Smith J.N., Dziuba N., Holbrook M.R., Zhang L., Kilpatrick J.M. (2008). Injectable peramivir mitigates disease and promotes survival in ferrets and mice infected with the highly virulent influenza virus, A/Vietnam/1203/04 (H5N1). Virology.

[27] Dillon R.C., Witcher R., Cies J.J., Moore W.S., Chopra A. (2017). Pharmacokinetics of Peramivir in an Adolescent Patient Receiving Continuous Venovenous Hemodiafiltration. J. Pediatr. Pharmacol. Ther..

[28] Koshimichi H., Tsuda Y., Ishibashi T., Wajima T. (2019). Population Pharmacokinetic and Exposure-Response Analyses of Baloxavir Marboxil in Adults and Adolescents Including Patients With Influenza. J. Pharm. Sci..

[29] Reed L.J., Muench H. (1938). A simple method of estimating fifty per cent endpoints. Am. J. Epidemiol..

[30] McKimm-Breschkin J.L., Jiang S., Hui D.S., Beigel J.H., Govorkova E.A., Lee N. (2018). Prevention and treatment of respiratory viral infections: Presentations on antivirals, traditional therapies and host-directed interventions at the 5th ISIRV Antiviral Group conference. Antiviral. Res..

[31] Omoto S., Speranzini V., Hashimoto T., Noshi T., Yamaguchi H., Kawai M., Kawaguchi K., Uehara T., Shishido T., Naito A. (2018). Characterization of influenza virus variants induced by treatment with the endonuclease inhibitor baloxavir marboxil. Sci. Rep..

[32] Philippa J., Baas C., Beyer W., Bestebroer T., Fouchier R., Smith D., Schaftenaar W., Osterhaus A. (2007). Vaccination against highly pathogenic avian influenza H5N1 virus in zoos using an adjuvanted inactivated H5N2 vaccine. Vaccine.

[33] Noshi T., Sato K., Ishibashi T. Pharmacokinetic and pharmacodynamic analysis of S-033188/S-033447, a novel inhibitor of influenza virus Cap-dependent endonuclease, in mice infected with influenza A virus [P1973]. Proceedings of the Final Programme of the 27th European congress of clinical Microbiology and Infectious Diseases.

[34] Bean B., Moore B.M., Sterner B., Peterson L.R., Gerding D.N., Balfour H.H. (1982). Survival of Influenza Viruses on Environmental Surfaces. J. Infect. Dis..

[35] Wood J.P., Choi Y.W., Chappie D.J., Rogers J.V., Kaye J.Z. (2010). Environmental Persistence of a Highly Pathogenic Avian Influenza (H5N1) Virus. Environ. Sci. Technol..

[36] Yamamoto Y., Nakamura K., Yamada M., Mase M. (2010). Persistence of avian influenza virus (H5N1) in feathers detached from bodies of infected domestic ducks. Appl. Environ. Microb..

[37] Gubareva L.V., Mishin V.P., Patel M.C., Chesnokov A., Nguyen H.T., De La Cruz J., Spencer S., Campbell A.P., Sinner M., Reid H. (2019). Assessing baloxavir susceptibility of influenza viruses circulating in the United States during the 2016/17 and 2017/18 seasons. Eurosurveillance.

